# Effects of Premedication With Midazolam on Recovery and Discharge Times After Tonsillectomy and Adenoidectomy

**DOI:** 10.7759/cureus.13101

**Published:** 2021-02-03

**Authors:** Andrew Garcia, Elizabeth A Clark, Sohel Rana, Diego Preciado, George M Jeha, Omar Viswanath, Ivan Urits, Alan D Kaye, Claude Abdallah

**Affiliations:** 1 Anesthesiology, George Washington University School of Medicine and Health Sciences, Washington, D.C., USA; 2 Anesthesiology, Children’s National Medical Center, Washington, D.C., USA; 3 Otolaryngology - Head and Neck Surgery, Children’s National Medical Center, Washington, D.C., USA; 4 Anesthesiology, Louisiana State University Health Sciences Center, New Orleans, USA; 5 Pain Management, Valley Pain Consultants Envision Physician Services, Phoenix, USA; 6 Anesthesia, Critical Care and Pain Medicine, Beth Israel Deaconess Medical Center, Harvard Medical School, Boston, USA; 7 Anesthesiology, Louisiana State University Health Sciences Center, Shreveport, USA

**Keywords:** midazolam, anesthesia, tonsillectomy, adenoidectomy, apnea

## Abstract

Background

Midazolam is commonly used preoperatively for anxiety. Adverse effects data in pediatric patients with obstructive sleep apnea (OSA) undergoing tonsillectomy and adenoidectomy (T&A) is limited.

Aims

We hypothesized that preoperative midazolam increases the time to emergence from anesthesia and postoperative discharge. Secondary objectives assessed if patients receiving midazolam experienced increased side effects or complications from treatment.

Methods

This study was a retrospective chart review of patients undergoing T&A from July 2014 to December 2015. Midazolam receiving patients (midazolam group: MG) were compared to patients who did not (non-midazolam group: NMG). Multivariable analyses were performed and adjusted for predefined potential cofounder variables.

Results

Emergence and discharge times were 5.2 minutes (95% CI [-7.1, 17.4]; p=0.41) and 10.1 minutes (95% CI [-6.7, 26.8]; p=0.24) longer in MG. These results were not statistically significant. Comparing by OSA status, there was no statistical difference in emergence and discharge times between mild, moderate and severe OSA groups or between MG and NMG within each OSA group. Emergence and discharge times in moderate OSA was 6.1 minutes (95% CI [-17.6, 29.8]; p=0.61) and 18.8 minutes (95% CI [-16.4, 53.9]; p=0.29) longer than mild OSA, and in the severe OSA group, 2.6 minutes (95% CI [-19.9, 25.1]; p=0.82) shorter and 2.8 minutes (95% CI [-30.3, 35.9]; p=0.87) longer. The incidence of postoperative complications was comparable between MG and NMG groups.

Conclusions

Premedication with midazolam was not associated with prolonged emergence or discharge time or higher incidence of complications after anesthesia for T&A in patients with OSA.

## Introduction

Over 289,000 ambulatory tonsillectomies are performed each year in children under 15 years of age, making this the second most common pediatric surgical procedure in the United States [1]. The two main indications for the procedure are recurrent throat infections and obstructive sleep-disordered breathing (OSDB). Obstructive sleep apnea (OSA) is a subcategory of OSDB that is defined as OSDB with an obstructive apnea-hypopnea index (AHI) of ≥ 1 seen during polysomnography (PSG). OSA is a relatively common problem estimated to impact between 1.2% and 5.7% of children [1]. In pediatric populations, OSA can result in poor school performance, failure to thrive, and significantly lower quality of life scores in several subscales such as general health, behavior, and physical functioning [1,2]. In order to mitigate the adverse effects of OSA on pediatric health and quality of life, tonsillectomy and adenoidectomy (T&A) is often indicated [1].

Midazolam is a benzodiazepine, commonly administered as pre-procedural anxiolytic in pediatric patients undergoing surgery. While it is common for children to experience preoperative anxiety, it is paramount to control this sensation, as perioperative anxiety is predictive of negative clinical outcome. Children who are more anxious preoperatively may experience significantly more pain postoperatively. This can lead to greater pain medication consumption, emergence delirium, and postoperative sleep problems [3].

The most prominent adverse reactions of concern in the perioperative period in children secondary to midazolam administration are apnea, bradypnea, paradoxical reactions, and possible postoperative delirium [4]. There is a concern for worsened upper airway collapse and less favorable patient outcomes in pediatric patients with OSA receiving midazolam prior to T&A, especially with concomitant use of opioids or other sedative medications such as dexmedetomidine or propofol.

In order to assess the potential impact of premedication with midazolam in pediatric patients with OSA undergoing tonsillectomy and adenoidectomy, we investigated both the impact of midazolam premedication on recovery time from general anesthesia, as well as the influence of patients’ AHI, a key indicator of OSA severity, on the emergence from general anesthesia and discharge time and quality, in pediatric patients with known OSA after T&A.

## Materials and methods

Study participants

This study included an electronic chart review of 524 patients at Children’s National Medical Center in Washington, DC, who underwent a T&A procedure between July 2014 and December 2015. Patients analyzed in this study were between the ages of 1 and 9 years. Information gathered included demographics: age, weight, body metabolic index (BMI), gender, race/ethnicity, AHI score, American Society of Anesthesia (ASA) physical status classification score, date of admission/discharge, diagnosis, length of surgery, anesthesia: including medications used in the preoperative and postoperative period, postoperative complications, and Aldrete recovery score.

Subjects were stratified based on whether they had received midazolam as part of their preoperative anesthesia regimen, as well as by OSA status for pediatric patients based on International Classification of Sleep Disorders Criteria (mild OSA = 1-5 apneic events per hour, moderate OSA = 5-10, severe OSA = >10). In total, 470 out of 524 patients whose charts had been reviewed met inclusion criteria and were included in the analysis. Demographic characteristics are detailed in Table 1.

**Table 1:** Demographic characteristics by midazolam status. *p-values were obtained from t-test for continuous data, and Chi-square test for categorical data. **ASA score (1 = a normal healthy patient; 2 = a patient with mild systemic disease; 3 = severe systemic disease). ASA: American Society of Anesthesia.

Characteristics	Overall	Midazolam		p-value*
	(N = 470)	No (n = 387)	Yes (n =83)	
Age, mean (SD)	3.6 (1.4)	3.7 (1.4)	3.4 (1.4)	0.14
Weight, mean (SD)	17.2 (7.6)	17.4 (7.8)	16.5 (6.3)	0.31
BMI, mean (SD)	16.9 (3.9)	16.9 (3.9)	16.8 (3.9)	0.88
Gender				
Male	263 (56.0%)	212 (54.8%)	51 (61.4%)	0.27
Female	207 (44.0%)	175 (45.2%)	32 (38.6%)	
Race				
African American/Black	216 (46.1%)	187 (48.4%)	29 (34.9%)	0.16
Caucasian	84 (17.9%)	66 (17.1%)	18 (21.7%)	
Hispanic/Latino	116 (24.7%)	92 (23.8%)	24 (28.9%)	
Other	53 (11.3%)	41 (10.6%)	12 (14.5%)	
Extubation:				
Awake	79 (16.8%)	68 (17.6%)	11 (13.3%)	0.34
Deep	391 (83.2%)	319 (82.4%)	72 (86.7%)	
ASA score**				
1	70 (14.9%)	56 (14.5%)	14 (16.9%)	0.41
2	360 (76.8%)	295 (76.4%)	65 (78.3%)	
3	39 (8.3%)	35 (9.1%)	4 (4.8%)	

Inclusion/exclusion criteria

Patients were included in the study if diagnosed with OSA, within nine years of age at the time of procedure, undergoing T&A under general anesthesia with endotracheal intubation. Patients were also included if they met ASA physical status score of 1-3. This score assesses the health fitness of patients before surgery, with a score of 1 meaning a patient with no systemic illness, a score of 2 indicating a mild systemic disturbance without substantive functional limitations, and a score of 3 indicating severe systemic disease that is not life-threatening.

Patients were excluded if they did not meet the above criteria and if they had any severe systemic organ disease (cardiopulmonary/renal/liver), neurological disease, or receiving antiseizure medications or any medication with possibility of interfering with midazolam pharmacokinetics or pharmacodynamics such as antifungals, macrolide antibiotics, cimetidine, rifamycins or calcium channel blockers.

Study design

This study was a retrospective chart review of patients at Children’s National Hospital who underwent T&A between July 2014 and December 2015. The project was approved by the Institutional Review Board at Children’s National Hospital. Information was gathered from electronic medical and anesthesia records and saved into a password protected Excel file kept offline, with access limited to investigators only.

Objective

The major objective of this study was to determine the effect of oral premedication with midazolam on recovery and discharge times from general anesthesia after T&A in patients with OSA. We hypothesized that preoperative midazolam may increase the time to emergence and discharge in pediatric patients with mild, moderate and severe OSA.

Primary endpoint

The primary endpoints included emergence and discharge times for individual patients. Information used to determine the primary endpoint was derived from the anesthesia and surgical records, including awake versus deep (prior to gaining consciousness) endotracheal extubation, fraction of inspired oxygen (FiO2) change, and length of surgery. Emergence time was calculated from the time the FiO2 increased by 10% at the end of surgery to tracheal extubation in the case of awake tracheal extubation, and as the time recorded for eye-opening by the postoperative anesthesia care unit (PACU) nurse in case of deep tracheal extubation. The increase in FiO2 marks the end of surgery and beginning of emergence, knowing that the FiO2 is kept low (<35%) during surgery to avoid airway fire, and it is increased as soon as the surgery ends in order to begin the process of anesthesia emergence. Discharge time was defined as the time the Aldrete recovery score of 10 was recorded by the PACU nurse. This score is used to determine whether a patient is safe to be discharged from PACU, assigning a 0-, 1-, or 2-point score based on consciousness, mobility, breathing, circulation, and color.

Secondary endpoint

Secondary endpoints included analyzing complications and the effect of degree of severity of apnea (as determined by AHI) on the patients’ emergence from general anesthesia and discharge after surgery and anesthesia recovery. Desaturations (SpO2<92%) and apnea episodes in the perioperative period (brief apnea <15 sec; prolonged apnea ≥15 sec or if associated with bradycardia), nausea and vomiting as well as delirium and need for supplemental pain medication in the postoperative period were also analyzed.

Statistical analysis

Descriptive statistics for patient demographic and baseline data were presented as mean with standard deviation for continuous variables and frequencies with percentages for categorical variables. Background characteristics and intraoperative medication dose between MG and NMG were compared using two sample t-test for continuous data and Chi-square test for categorical data. BMI between the three OSA groups was compared using a one-way analysis of variance (ANOVA) test.

We used multiple linear regression to compare our primary endpoints emergence time and discharge time between MG and NMG as well as between mild, moderate and severe OSA groups. All multivariable analyses were performed adjusting for predefined potential cofounder variables such as surgery duration, intraoperative dexmedetomidine, propofol, and fentanyl dose, and awake or deep tracheal extubation status that could potentially influence the outcomes. Normality assumptions were tested using Shapiro-Wilks test as well as graphical assessments (histogram and qq plot). All reported p values were two-sided. Our study had a statistical power of 95% to detect a medium effect size (d=0.5) in comparing mean emergence and discharge time between two groups using a two-sided two-sample equal-variance t-test with a significance level (alpha) 0.05. Power analysis was done using PASS 2020 software and all other statistical analyses were performed with Stata software, version 15.1 MP [5,6].

## Results

A total of 470 patients were retained in the study from the 524 patients who underwent T&A during the period of the study. The reason for exclusion of 54 patients was not meeting inclusion criteria and/or deficiency in electronic charting. The average age of the patients in the sample was 3.6 years (±1.4 years) ranging from 0.75 to 8.5 years. 44% of the patient population was female, 46.1% of the patients were African Americans. Detailed patient demographics are shown in Table 1.

Of the 470 patients included in the analysis, 83 (17.7%) received midazolam and 387 (82.3%) did not. When stratified by whether midazolam was received or not, there were no significant differences in patient demographics found between the two groups. The mean midazolam dose was 0.54mg/kg (SD: 0.12), and there was no dose-dependent response relationship between emergence and discharge times and midazolam dose in the MG. We compared intraoperative medication dose (fentanyl, dexmedetomidine, and propofol) between the two groups, and both groups were similar in terms of intraoperative medication except for fentanyl, which was 0.2 mcg/kg higher in the NMG (p=0.042) (Table 2).

**Table 2:** Mean intraoperative medication dose by midazolam status. *p-values were obtained from unpaired t-test. **Dose reported as mean (standard deviation).

Medications	Midazolam		p-value*
	No (n = 387)	Yes (n = 83)	
Fentanyl (mcg/kg)	0.7 (0.7)	0.5 (0.7)	0.042
Dexmedetomidine (mcg/kg)	1.0 (0.8)	1.1 (0.9)	0.31
Propofol (mg/kg)	38.6 (24.2)	37.5 (26.2)	0.75

The mean duration of the procedure was 17.5 (SD: 11.2) minutes in MG and 16.9 (SD: 9.4) minutes in NMG and there was no statistical difference (p=0.64) between the MG and NMG.

**Figure 1:**
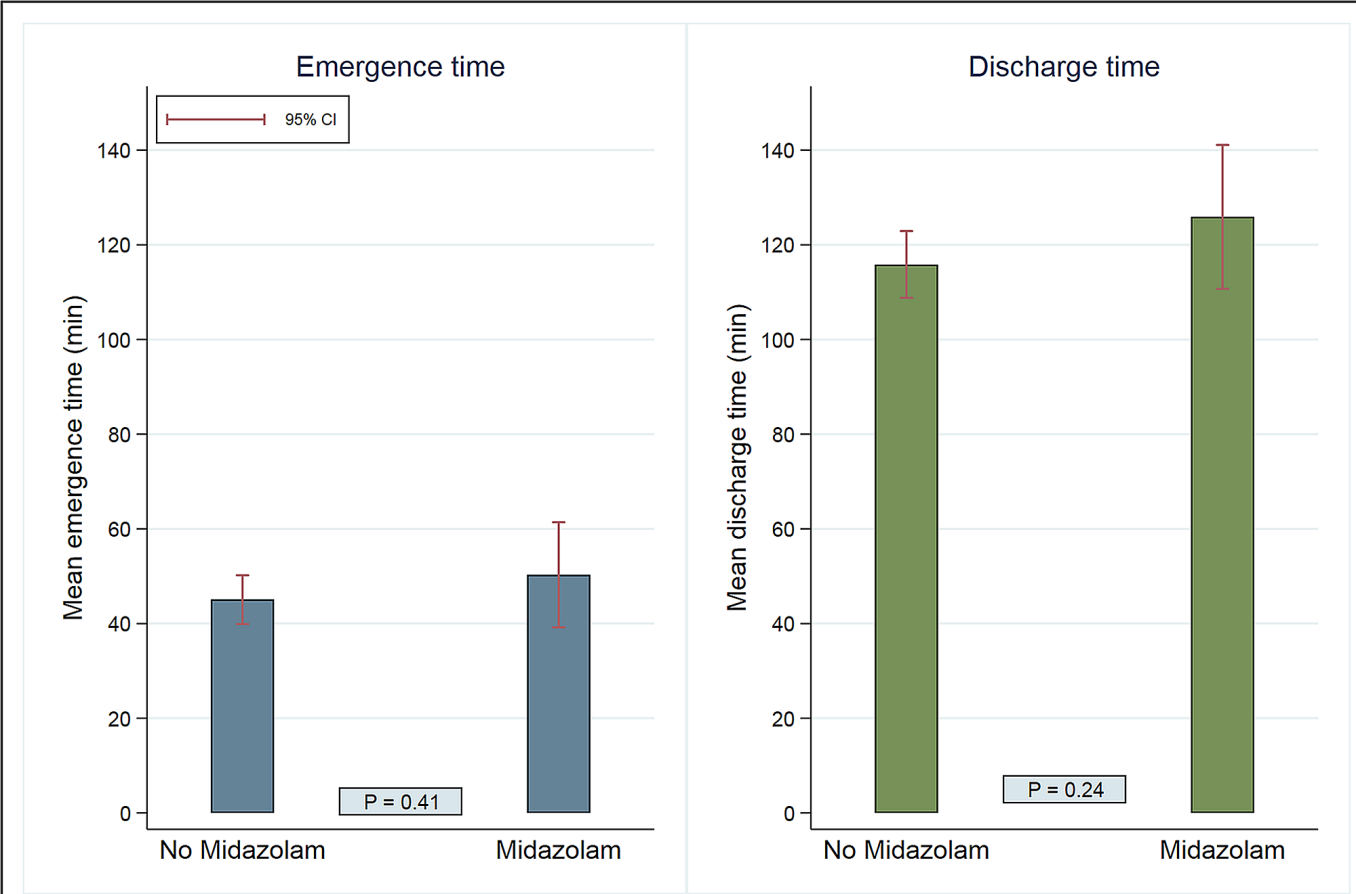
Comparison of emergence time and discharge time between midazolam and no midazolam group.

After adjusting for all potential confounders (surgery duration, propofol, dexmedetomidine, and fentanyl doses adjusted to weight, and awake or deep tracheal extubation status), emergence time was found to be 5.2 minutes (95% CI [-7.1, 17.4]; p=0.41) longer in MG (50.3 minutes, 95% CI [39.2, 61.4]) comparing to NMG (45.1 minutes, 95% CI [39.9, 50.2]). Discharge time was found to be 10.1 minutes (95% CI [-6.7, 26.8]; p=0.24) longer in the MG (125.9 minutes, 95% CI [110.7, 141.1]) comparing to the NMG (115.8 minutes, 95% CI [108.8, 122.9]). None of these differences was statistically significant (Figure 1).

**Figure 2:**
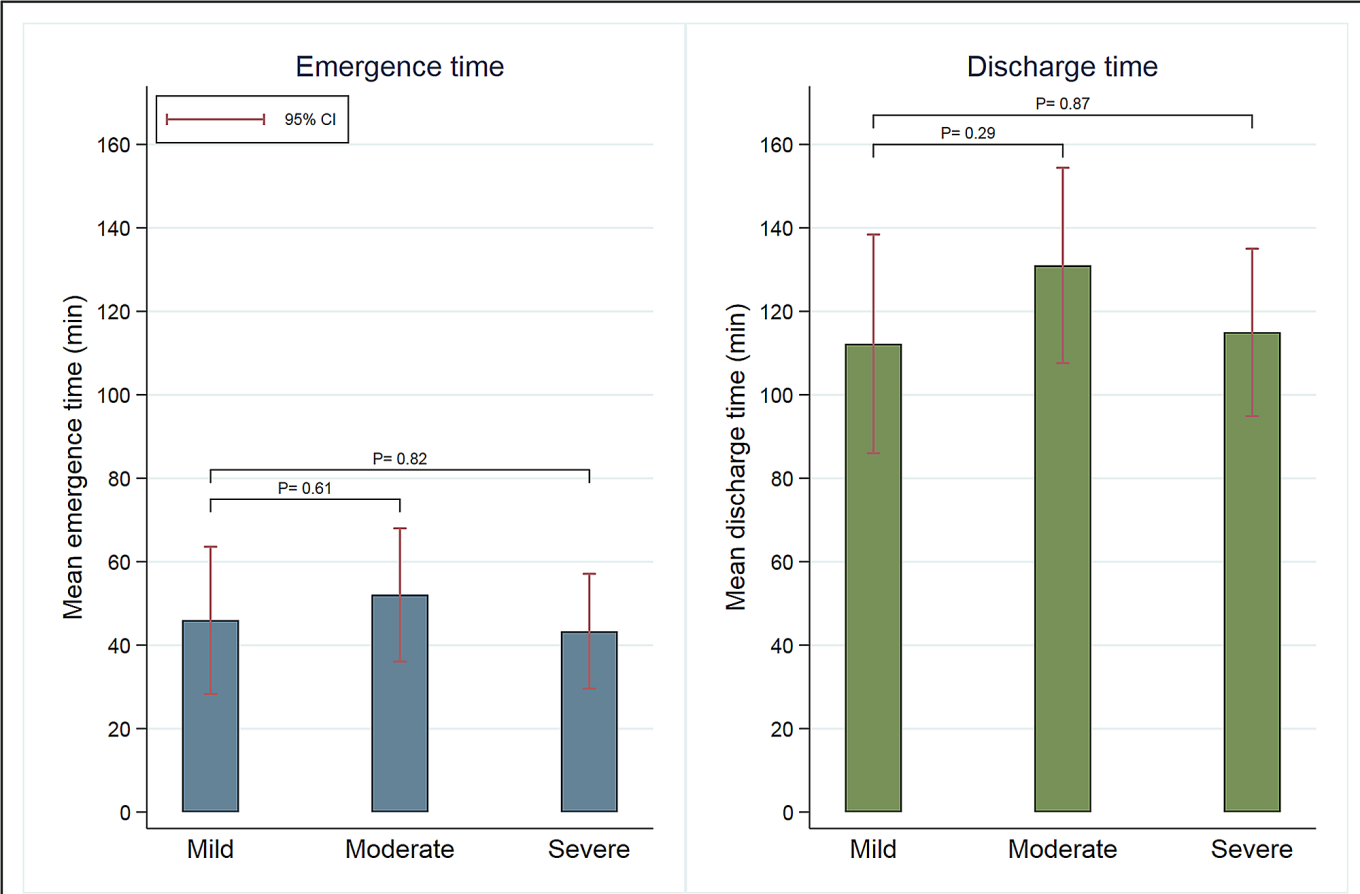
Comparison of emergence time and discharge time between mild, moderate, and severe OSA group. OSA: obstructive sleep apnea.

We performed a sub-analysis of emergence and discharge times by OSA status among patients with AHI data available (n=133). In this analysis, there were 33 patients with mild OSA, 42 patients with moderate OSA, and 58 patients with severe OSA. An analysis of variance showed that there was no difference in BMI between the three OSA groups, (F (2, 127) = 0.85; p=0.429). For emergence time stratified by OSA status, mild OSA served as a reference, with mean emergence time of 46 minutes (95% CI [28.3, 63.6]). Moderate OSA mean emergence time was 52.1 minutes (95% CI [36.1, 68.0]) and severe OSA mean emergence time was 43.3 minutes (95% CI [29.6, 57.1]). For mean discharge time, mild OSA served as the reference, with a mean discharge time of 112.2 minutes (95% CI [86.0, 138.4]). The mean discharge time for moderate OSA was 131 minutes (95% CI [107.6, 154.4]). Compared to mild OSA, emergence time and discharge time in the moderate OSA group was 6.1 minutes (95% CI [-17.6, 29.8]; p=0.61) and 18.8 minutes (95% CI [-16.4, 53.9]; p=0.29) longer respectively, and in the severe OSA group, 2.6 minutes (95% CI [-19.9, 25.1]; p=0.82) shorter and 2.8 minutes (95% CI [-30.3, 35.9]; p=0.87) longer respectively. None of these differences were statistically significant (Figure 2). 

Furthermore, there was no significant statistical difference in discharge time and emergence time between the MG group and NMG within each OSA group (Table 3).

**Table 3:** Comparison of emergence and discharge times between midazolam and no midazolam group within each OSA group. OSA: obstructive sleep apnea.

OSA group	Mean emergence time (95% CI), min			Difference (95% CI), min	p-value
	Midazolam group		No Midazolam group		
Mild	31.2 (0.0, 73.1)		48.7 (29.1, 68.4)	-17.5 (-63.7, 28.7)	0.46
Moderate	46.0 (9.7, 82.3)		52.8 (35.0, 70.6)	-6.8 (-47.2, 33.5)	0.74
Severe	33.7 (0, 80.9)		45.0 (30.7, 59.3)	-11.3 (-60.4, 37.8)	0.65
OSA Group	Mean discharge time (95% CI), min			Difference (95% CI), min	p-value
	Midazolam group	No Midazolam group			
Mild	143.5 (81.5, 205.4)	105.3 (76.3, 134.4)		38.1 (-30.3, 106.4)	0.27
Moderate	141.8 (88.1, 195.5)	126.6 (100.2, 152.9)		15.2 (-44.5, 74.9)	0.62
Severe	123.5 (53.7, 193.3)	115.3 (94.1, 136.5)		8.2 (-64.4, 80.8)	0.82

For secondary endpoints of desaturations and apnea in the perioperative period and delirium in the postoperative period, there were two apnea events, six desaturation episodes, and one delirium event observed, all from the NMG. For the apnea and delirium event, these occurred in patients who had a desaturation episode, indicating that seven patients total experienced adverse events. Characteristics of those patients who experienced events are listed in Table 4.

**Table 4:** Characteristics of patients experiencing adverse effects in study (all non-midazolam group). *Same patient; **same patient; ***from start of surgery. B: Black; C: Caucasian; O: other race; H: Hispanic.

	Age (years)	Weight (kg)	ASA	OSA severity	BMI	Gender	Race/ethnicity	Extubation type	Fentanyl dose	Timing of event (min)***
Apnea 1*	1.3	9.4	2	Not listed	16.3	F	B	Deep	0.53	Not listed
Apnea 2	3.3	11.2	2	Not listed	14.8	M	B	Deep	0	119
Desaturation 1*	1.3	9.4	2	Not listed	16.3	F	B	Deep	0.53	51
Desaturation 2**	6.2	55.2	3	Not listed	34.8	M	B	Deep	0.27	70
Desaturation 3	2.5	13.1	3	Not listed	16.9	M	C	Deep	1.91	1
Desaturation 4	3.0	13.3	2	5.2	16.1	M	OR	Deep	0	54
Desaturation 5	1.7	13.8	2	1.2	21.6	F	H	Deep	0	35
Desaturation 6	3.3	12.7	2	Not listed	15.3	M	B	Deep	0	58
Delirium 1**	6.2	55.2	3	Not listed	34.8	M	B	Deep	0.27	70

## Discussion

Midazolam is useful in procedural sedation and anxiolysis due to its distinct pharmacological properties. It acts within the central nervous system by binding to γ-aminobutyric acid (GABA-A) receptors of postsynaptic neurons, making them more permeable to chloride ions and inducing hyperpolarization and decreased excitability of the neuronal membrane, leading to the sedative and anticonvulsant effects of the medication [4]. Intravenously (IV), midazolam has an onset of 2-3 minutes with duration of action of approximately 45-60 minutes. The preferred and most tolerated route of administration for midazolam as a premedication is orally, to relieve anxiety prior to anesthesia induction and obtaining intravenous access in the majority of outpatients. The onset of action of oral midazolam is 10 to 20 minutes, with duration of action of 60-90 minutes, and a bioavailability of ~36% in children [4,7]. Administered oral doses may vary from 0.25-0.75 mg/kg; with 0.5 mg/kg being the most commonly administered dose in daily practice. Other routes of administration currently available, but less tolerated by children include intramuscularly (IM) and intranasally (NAS). In children, the half-life of midazolam is 2.9-4.5 hours IV and 2.2-6.8 hours PO. Clearance is 3.2-13.3 mL/kg/minute in children over the age of one year [4]. Midazolam is metabolized to its active metabolite by CYP3A4 (and to a lesser extent CYP2B6) and is ~97% distributed bound to albumin [4]. Adverse events such as oxygen desaturation, respiratory depression, apnea and airway obstruction have been reported in less than 1% of pediatric patients and are believed to be dose-dependent. Furthermore, paradoxical reactions such as inconsolable crying and delirium have been reported in 1%-15% of children receiving midazolam [7,8].

Earlier trials found that effects of midazolam on upper airway obstruction were most pronounced in two settings: when being when used with other respiratory depressing drugs such as opioids; and when used in patients with severe underlying respiratory disease or mechanical obstruction, such as severe OSA [9]. Patients with OSA are at an increased risk of upper airway collapse as a result of worsened pharynx obstruction after administration of sedatives, anesthetics, and analgesics. This is thought to be related to decreased pharyngeal muscle tone and diminished respiratory drive, particularly when respiratory depressants are given together [10]. This being said, adding midazolam to fentanyl is practiced widely in children without anatomical defects as a useful means of fast-acting analgesia and sedation with an acceptable side effect profile as long as both drugs are carefully titrated to effect [7].

The influence of other anesthetic drugs on recovery from midazolam has also been investigated. Viitanen et al found that midazolam premedication delayed recovery but not discharge from propofol-induced sevoflurane anesthesia in children 1-3 year [11]. The patients in this study received atropine and alfentanil in addition to propofol, tracheal intubation was facilitated with mivacurium, and anesthesia was maintained with nitrous oxide/oxygen and sevoflurane with controlled ventilation. Propofol, a commonly used intravenous anesthetic for the induction of anesthesia, is GABA mediated, like midazolam. It acts as a GABAA receptor positive allosteric modulator, and at high doses as a receptor agonist [12]. For pediatric procedural sedation, concomitant use of midazolam with propofol may increase serum concentrations of both drugs [4,7,13]. Propofol reduces the distribution and clearance of midazolam in a concentration-dependent manner, and midazolam reduces the metabolic and rapid and slow distribution clearances of propofol [4,14,15]. With regards to the sevoflurane and nitrous oxide used in the Viitanen et al study, these agents have not been observed to affect the awakening or discharge times in pediatric patients receiving oral midazolam 0.5 mg/kg as premedication when compared to placebo [16].

Although there are guidelines available from the American Society of Anesthesiologists regarding the perioperative management of OSA patients and general risks of respiratory depression and airway collapse with sedatives and opioids, guidance for using specific agents like midazolam in pediatric patients is limited [17]. Many institutions have created their own guidelines for sedative use in children at their hospitals. An online survey of 110 pediatric anesthesiologists conducted by Roberts et al. indicated that 27.3% of respondents reported that their institutions had such guidelines for perioperative management of children with OSA undergoing T&A, and that while 53.6% of respondents administered oral midazolam preoperatively in patients with severe OSA, 24.5% typically withheld the premedication [18].

In this study, we aimed to determine the effect of oral premedication with midazolam on recovery from general anesthesia after T&A in pediatric patients with OSA, as well as determine differences in complications such as perioperative desaturation and apnea, and postoperative delirium, hypothesizing that preoperative midazolam would increase time to emergence and discharge in pediatric patients with mild, moderate and severe OSA.

Primary endpoint: emergence and discharge

We did not find any significant differences between the MG and NMG groups with regards to the emergence and discharge endpoints, leading us to reject our hypothesis. This study represents an ongoing effort to best address the question of appropriateness of preoperative midazolam in pediatric patients with OSA undergoing surgical procedures.

In 2002, Cultrara et al conducted a retrospective study of 65 patients with a clinical diagnosis of OSA and found no difference in adverse events defined by upper airway obstruction (hypoventilation, desaturation, bradycardia, or sustained lethargy) within 24 hours after surgery when a mean preoperative midazolam dose of 0.4 mg/kg was given [10]. A prospective observational study by Francis et al in 2006 to detect respiratory compromise in 70 children receiving midazolam 0.5 mg/kg prior to T&A determined that only two patients (2.9%) in their sample developed measurable adverse effects related to administration of midazolam, ultimately concluding that, for patients over the age of 3 and without the presence of severe OSA, there was a low level of morbidity associated with midazolam premedication [19]. They recommended that for patients with severe OSA, a reduced dose should be considered or an alternative medication with minimal respiratory side effects should be used, such as clonidine or dexmedetomidine. A study by Du et al even found that a single preoperative dose of dexmedetomidine may provide better postoperative anxiolytic effects than midazolam in a 2019 study [20].

Our findings showed no difference in recovery and discharge from anesthesia. With a mean dose of 0.54mg/kg, (SD: 0.12), there appeared to be a trend of longer times in the MG for primary endpoints, but not enough of a difference to be statistically significant. The trend of shorter rather than longer discharge times in the severe OSA group, while unexpected, may be explained by the possible avoidance of pediatric anesthesiologists to administer this premedication in this category of patients. In case of short surgical procedures such as T&A, the effect of midazolam may be persistent throughout the recovery period. It appears that there was a careful titration of intravenous fentanyl in patients who received midazolam, which would explain the statistically significant difference between the dose of fentanyl in the MG and NMG. This would have prevented the additive effect of these medications on respiratory depression and further complications. Previous research had demonstrated that reduced opioid requirement for analgesia in children displaying oxygen desaturation associated with severe OSA may be related to their young age and to an up-regulation of central opioid receptors consequent to recurrent hypoxemia [21,22]. Midazolam premedication delays recovery from propofol-induced sevoflurane anesthesia in children 1-3 years.

Secondary endpoint: complications

It was unexpected to observe that the incidence of adverse effects such as apneas and desaturation events was less common in MG versus NMG, given the extra risk factor for respiratory depression in MG. The fact that most patients in our study did not receive midazolam if they had severe OSA, may have accounted for a lack of increased incidence, as they may have been considered too high risk for this medication [4]. A common factor in patients who experienced documented complications (apnea/desaturations after surgery) as described in Table 4 is deep tracheal extubation. This finding may have been expected since while advantages of tracheal extubation under deep anesthesia include reducing the incidence of immediate coughing and therefore straining on the endotracheal tube and cardiovascular stimulation; the respiratory complications, especially in PACU, may be higher with deep endotracheal extubation regardless of the type of surgery [23]. Emergence delirium has been documented after oral midazolam administration in children in previous studies [24,25]. Vittanen et al documented that more children premedicated with midazolam suffered from arousal distress and scored higher on the Pain/Discomfort scale after arrival in the recovery room [24]. Unlike that study; however, none of our patients received muscle relaxation to facilitate tracheal intubation and nitrous oxide was not used for maintenance of anesthesia. In our study, emergence delirium did not seem to be increased in the MG.

Limitations/weaknesses

We were able to include a large enough effect size to achieve an appropriately powered study. This study demonstrated that there was no significant statistical difference in emergence and discharge times between MG and NMG; however, this difference may present a significant and valuable occupation of the recovery room that has not been calculated, since this was not the objective of this study. A future prospective study restricted to patients with documented OSA as per sleep study may be able to delineate the best management of these patients; however, our study reflects the actual clinical daily practice and perioperative management of pediatric patients with OSA in a specialized pediatric hospital

## Conclusions

Premedication with midazolam was not associated with a prolonged emergence or discharge time or with a higher incidence of complications after anesthesia for tonsillectomy and adenoidectomy in patients with obstructive sleep apnea. These results may not be extrapolated to different dosages of midazolam or combination of medications including narcotics/hypnotic medications. Close supervision and monitoring of surgical pediatric patients with OSA are always recommended. More research is needed in order to characterize the optimal perioperative management in pediatric patients with obstructive sleep apnea undergoing surgery.
